# A novel gelatin/chitooligosaccharide/demineralized bone matrix composite scaffold and periosteum-derived mesenchymal stem cells for bone tissue engineering

**DOI:** 10.1186/s40824-021-00220-y

**Published:** 2021-06-16

**Authors:** Thakoon Thitiset, Siriporn Damrongsakkul, Supansa Yodmuang, Wilairat Leeanansaksiri, Jirun Apinun, Sittisak Honsawek

**Affiliations:** 1 Biomedical Engineering Program, Faculty of Engineering, Chulalongkorn University, Bangkok, 10330 Thailand; 2 Department of Chemical Engineering, Biomaterial Engineering for Medical and Health Research Unit, Faculty of Engineering, Chulalongkorn University, Bangkok, 10330 Thailand; 3 Research Affairs, Faculty of Medicine, Chulalongkorn University, Excellence Center for Advanced Therapy Medicinal Products, King Chulalongkorn Memorial Hospital, Bangkok, 10330 Thailand; 4 School of Preclinic, Institute of Science, Suranaree University of Technology, 111 University Avenue, Muang, Nakhon Ratchasima, 30000 Thailand; 5 Department of Orthopaedics, Vinai Parkpian Orthopaedic Research Center, Faculty of Medicine, Chulalongkorn University, Bangkok 10330, Thailand; 6 Department of Biochemistry, Osteoarthritis and Musculoskeleton Research Unit, Faculty of Medicine, Chulalongkorn University, Rama IV road, Pathumwan, Bangkok, 10330 Thailand

**Keywords:** Chitooligosaccharide, Demineralized bone, Gelatin, Mesenchymal stem cells, Tissue engineering

## Abstract

**Background:**

A novel biodegradable scaffold including gelatin (G), chitooligosaccharide (COS), and demineralized bone matrix (DBM) could play a significant part in bone tissue engineering. The present study aimed to investigate the biological characteristics of composite scaffolds in combination of G, COS, and DBM for in vitro cell culture and in vivo animal bioassays.

**Methods:**

Three-dimensional scaffolds from the mixture of G, COS, and DBM were fabricated into 3 groups, namely, G, GC, and GCD using a lyophilization technique. The scaffolds were cultured with mesenchymal stem cells (MSCs) for 4 weeks to determine biological responses such as cell attachment and cell proliferation, alkaline phosphatase (ALP) activity, calcium deposition, cell morphology, and cell surface elemental composition. For the in vivo bioassay, G, GC, and GCD, acellular scaffolds were implanted subcutaneously in 8-week-old male Wistar rats for 4 weeks and 8 weeks. The explants were assessed for new bone formation using hematoxylin and eosin (H&E) staining and von Kossa staining.

**Results:**

The MSCs could attach and proliferate on all three groups of scaffolds. Interestingly, the ALP activity of MSCs reached the greatest value on day 7 after cultured on the scaffolds, whereas the calcium assay displayed the highest level of calcium in MSCs on day 28. Furthermore, weight percentages of calcium and phosphorus on the surface of MSCs after cultivation on the GCD scaffolds increased when compared to those on other scaffolds. The scanning electron microscopy images showed that MSCs attached and proliferated on the scaffold surface thoroughly over the cultivation time. Mineral crystal aggregation was evident in GC and greatly in GCD scaffolds. H&E staining illustrated that G, GC, and GCD scaffolds displayed osteoid after 4 weeks of implantation and von Kossa staining confirmed the mineralization at 8 weeks in G, GC, and GCD scaffolds.

**Conclusion:**

The MSCs cultured in GCD scaffolds revealed greater osteogenic differentiation than those cultured in G and GC scaffolds. Additionally, the G, GC, and GCD scaffolds could promote in vivo ectopic bone formation in rat model. The GCD scaffolds exhibited maximum osteoinductive capability compared with others and may be potentially used for bone regeneration.

## Introduction

Demineralized bone matrix (DBM) is well-known as an osteoinductive biomaterial utilized for tissue engineering. When DBM is combined with osteoprogenitors and implanted into animals, it could support and induce new bone formation in in vivo [1]. Moreover, DBM could trigger osteoinductivity both in vitro and in vivo. The osteoinductivity of the DBM may result from collagenous proteins and noncollagenous proteins, including basic fibroblast growth factor (bFGF), transforming growth factor-β (TGF-β), and especially bone morphogenetic proteins (BMPs) [2]. Alidadi and colleagues evaluated DBM combined with chitosan and polymethylmethacrylate in radial bone defect of rat model, showing that DBM provided significantly superior biocompatibility, biodegradability, osteoconductivity, and osteoinductivity to the scaffold [3]. Furthermore, DBM significantly improved bone healing close to the autologous cortical bone graft.

Gelatin is a highly biocompatible (low immunogenic capacity), biodegradable, noncytotoxic, and non-antigenic natural polymer that is derived from the hydrolytic process of collagen [4]. Moreover, gelatin comprises an arginine-glycine-aspartic acid (RGD) motif that supports cell adhesion, migration, and proliferation [4]. Previous studies demonstrated that gelatin could be used to produce three-dimensional scaffolds for bone tissue regeneration since its physicochemical properties can be properly manipulated [5]. Therefore, gelatin is selected as a base material to fabricate the scaffolds combined with collagen for bone tissue engineering.

Chitosan is a glycosaminoglycan (GAG) analog due to the similarity of its molecular structure to natural GAG [3]. Chitosan, the deacetylated polysaccharide of chitin, is an amino polysaccharide consisting of β-(1, 4)-2-acetamido-2-deoxy-D-glucose and β-(1, 4)-2-amino-2-deoxy-D-glucose units. It exhibits attractive biological properties, including antimicrobial activity, antitumor effects, and immunomodulatory biological assets [6]. Also, chitosan is documented to accelerate neovascularization and promote new bone formation [7]. Depending on source and preparation processes, the molecular weight of chitosan can be varied in a wide range. A recent study has unveiled that low-molecular-weight chitosan has higher effectiveness in stimulating the proliferation of mouse fibroblasts than high-molecular-weight chitosan [8].

Chitooligosaccharide (COS) is a very low-molecular-weight water-soluble chitosan that can be derived from various approaches such as physical method, chemical hydrolysis, and enzymatic degradation [9]. In recent years, polysaccharides-like chitosan and its derivatives have become of increasing interest in the field of tissue engineering [9]. Ratanavaraporn and colleagues unveiled that gelatin and chitooligosaccharide (G/COS) scaffolds enhanced the attachment, proliferation, and osteogenic differentiation of rat bone marrow-derived mesenchymal stem cells (MSCs) in vitro [10]. Importantly, G/COS scaffolds promoted a higher osteogenic differentiation ability of rat MSCs than pure gelatin scaffolds [10, 11]. Particularly, G/COS scaffolds in the blending ratio of 70/30 were found to enhance osteogenic differentiation of bone marrow-derived stem cells [11].

Apart from scaffold-based strategies, MSCs, another key element for bone tissue engineering, are promising cell sources for bone regeneration, capable of differentiating into various specialized mesenchymal tissues, including bone, tendon, cartilage, muscle, ligament, fat, and bone marrow. Stem cells have basically been seeded on scaffolds, cultured in vitro*,* and then implanted to observe bone formation in vivo. It is believed that stem cells can differentiate into osteoblasts and chondroblasts, which can then accelerate bone repair. In this study, the periosteum-derived MSCs are employed in vitro proliferation and osteogenic differentiation on the scaffolds.

Although DBM contains BMPs and osteoinductive proteins, it may be a challenge to fabricate into three-dimensional structures. Furthermore, gelatin and COS are alternative biomaterials used for tissue engineering that enhance osteogenic differentiation of bone marrow stromal cells. Recent studies reported that an addition of COS in the composite scaffold has shown to be more effective to promote and accelerate the adhesion, proliferation, and osteogenic differentiation of MSCs [10, 11]. Corresponding to existing literature, there have never been any studies describing tri-component bone scaffolds comprised of COS-gelatin matrix reinforced with DBM. In this study, it should be emphasized that the composition of fabricated biomaterials has characteristics of novelty. The simultaneous application of freeze-drying technique and cross-linking agent is simple and cost effective for the fabrication of the scaffolds imparting a highly porous structure with interconnected pores, which cannot be acquired by using merely freeze-drying or cross-linking processes. The feasibility to combine COS, gelatin, and DBM provides synergistic benefits of these biomaterials to formulate composite scaffolds for bone tissue engineering with desirable features such as biodegradability, biocompatibility, mechanical strength, and osteoinductivity.

As yet, novel scaffolds from natural biomaterials containing gelatin, COS, and DBM for bone tissue engineering have never been documented. Therefore, the objectives of this study were to develop novel gelatin/COS/DBM composite biomaterials for bone tissue engineering and to characterize their biological properties in three-dimensional biocomposite scaffolds for osteogenic differentiation of MSCs. Furthermore, the scaffolds were subcutaneously implanted into the back of Wistar rat to histologically determine the ectopic bone formation.

## Materials and methods

### Preparation of biomaterials

Demineralized bone matrix was prepared from bone allografts as previously described [12]. Concisely, the allograft bone was cleaned with 3% hydrogen peroxide and 70% isopropanol. The washed ground bone was pulverized into small particles, and the minerals were dissolved by an acid extraction process. Pulverized bone of less than 1000 μm particle size was selected for the next process. The bone was demineralized in 0.1 M HCl for 8 h with continuous stirring at 16 °C. The resulting powder was washed, lyophilized, and sieved into a particle size range of 250–500 μm. A gelatin (G) sample prepared by acidic treatment of porcine skin collagen (isoelectric point = 9) was kindly supplied by Nitta Gelatin, Osaka, Japan. Alaska crab water-soluble chitooligosaccharide (COS, MW = 2000 Da, deacetylation degree = 99%) was obtained from Hangzhou Garden Corporation, China. Other chemicals used were analytical and cell culture-tested grades.

### Fabrication of three-dimensional scaffolds

**Fig. 1:**
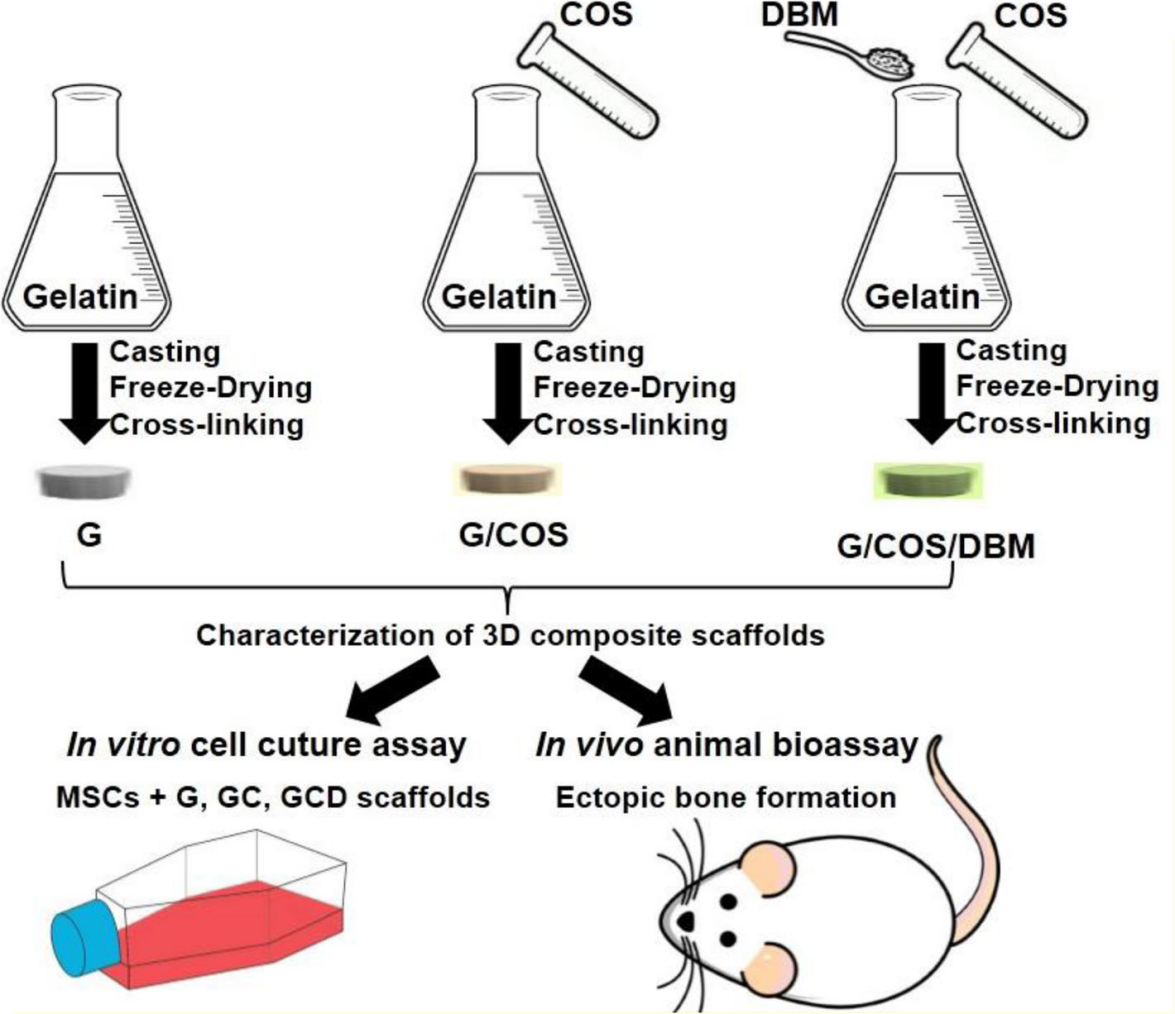
Schematic diagram of fabrication and characterization of G, GC, and GCD scaffolds

Three-dimensional scaffolds of G, G/COS (GC), and G/COS/DBM (GCD) were fabricated via freeze-drying and chemical crosslinking techniques (Fig. 1). Briefly, 2 wt% aqueous pure gelatin solution was prepared in 1% (v/v) acetic acid solution (pH 2.5) for 2 h at 60 °C. Next, the other blending ratio of gelatin to chitooligosaccharide (G/COS) 70/30 for 2 wt% aqueous solution was prepared in the same solvent and conditions. The dissolved solutions were added with 0.15% (v/v) glutaraldehyde solution and stirred for 15 min. The solutions were crosslinked under 4 °C for 12 h. The gelatin blended chitooligosaccharide (GC) solution 70/30 was added with demineralized bone powder in the blending ratio of 60/40 (GCD) and then mixed until homogeneous. All of the solutions were loaded into sterile 48-well culture plates and gradually frozen at -20 °C and -80 °C for 12 h. The freeze solution was freeze-dried using lyophilizer under vacuum for 24 h. The obtained scaffolds were immersed in 0.1 M glycine solution at room temperature for 2 h to block the non-reacted aldehyde groups and repeatedly washed with sterile deionized water before refreeze-drying. The scaffolds were sterilized before cultivation using ethylene oxide.

### Scanning electron microscopy

Scaffolds were cut horizontally with razor blades and sputter-coated with gold (Ion sputtering device, JFC 1100, Japan). Cross-sectional features of scaffolds were visualized by a scanning electron microscope (SEM, Jeol JSM 5400, JEOL, Japan) at an accelerating voltage of 12–15 kV after sputter-coating with gold. The pore diameter of the scaffold was analyzed from SEM micrographs via ImageJ Launcher program (*n* = 100).

### Porosity

Pore size of each scaffold was randomly analyzed from 100 pores/scaffold using ImageJ software (the US National Institutes of Health, USA). The porosity of all prepared samples was assessed by a liquid displacement method as described previously [13]. Hexane was used as the displacement liquid as it permeates through the scaffolds without swelling or shrinking the matrix. A known weight of dry scaffold was completely immersed in a known volume (V_1_) of hexane for 5 min. The volume of hexane and the hexane-impregnated scaffolds were recorded (V_2_). The hexane-impregnated scaffold was then removed and the residual volume of hexane was recorded (V_3_). Porosity was calculated using the following equation: scaffold porosity (%) = [(V_1_ − V_3_)/(V_2_ − V_3_)] × 100.

### Swelling ratio of the scaffolds

The initial dry scaffolds were weighed and recorded (W_D_). Swelling properties of the scaffolds were determined by their immersion in phosphate buffered saline (PBS, pH 7.4) for 24 h at the physiological temperature (37 °C). After the predetermined time period, the swollen scaffolds were removed from the medium and weighed (W_S_). The swelling ratio was calculated according to the following equation: swelling ratio = (W_S_ – W_D_)/W_D_.

### Compressive strength

A universal mechanical tester (Instron 5567, USA) was used to evaluate compressive modulus of scaffolds by compressing the scaffolds (13 mm in diameter and 3 mm in thickness) at a constant rate of 0.5 mm/min. The end point of compression was set at 50% of initial thickness of the scaffolds. The slopes of compressive stress–strain curves at 5–35% deformation were used to calculate the compressive modulus of the scaffolds.

### Isolation and culture of mesenchymal stem cells (MSCs)

The MSCs were isolated from human periosteum using primary outgrowth techniques [14]. The cells were incubated for 30 min at 4 °C with monoclonal antibodies against human anti-CD29, CD34, CD44, CD45, CD90, and CD105 conjugated with PE, FITC, APC, and PE/Cy5 (BioLegend, USA). Stained samples were analyzed using a flow cytometry (BD Biosciences, San Jose, CA, USA). As shown in a previous report [14], the periosteum-derived primary cells expressed MSC specific markers (CD29, CD44, CD90, CD105), while they did not express hematopoietic stem cell surface markers (CD34, CD45). Additionally, they exhibited the tri-lineage capacity to differentiate into osteogenic, chondrogenic, and adipogenic lineages and expressed marker genes for osteoblasts, adipocytes, and chondrocytes. The MSCs were cultured in alpha-minimum essential medium (α-MEM, Hyclone, Logan, UT, USA) supplemented with 10% fetal bovine serum (FBS, Hyclone, Logan, UT, USA) and 100 unit/ml penicillin/100 μg/ml streptomycin and incubated in a 5% CO_2_ incubator at 37 °C.

### Initial attachment and proliferation assay

MSCs (5.0 × 10^5^ cells/scaffold) were seeded onto the ethylene oxide-sterilized scaffolds and cultured in proliferating medium (α-MEM supplemented with 15% FBS) at 37 °C in a 5% CO_2_ incubator. The number of cells attached on the test scaffolds after 6 h seeding and cells proliferated on days 1, 3, 5, and 7 was determined using fluorometric quantification of cellular DNA according to the method reported by Takahashi [15]. Briefly, the cell-seeded scaffold samples were lysed in saline sodium citrate buffer (SSC, pH 7.4) containing sodium dodecyl sulfate at 37 °C overnight. Then, 100 μl of cell lysates were mixed with a fluorescent dye solution (Hoechst 33258 dye) in a 96-well black plate. Fluorescence intensities of the mixed solutions were spontaneously measured at the excitation and emission wavelengths of 355 and 460 nm, respectively. The standard curve between the DNA and cell number was prepared using standards of known cell numbers. The DNA assay was conducted three times independently for every experimental sample unless otherwise mentioned.

### Osteogenic differentiation of MSCs on scaffolds

MSCs (1.0 × 10^6^ cells/scaffold) were seeded on the scaffolds in proliferating medium under continuous shaking for 6 h. After seeding for 1 day, the medium was changed to osteogenic medium (α-MEM supplemented with 10% FBS, 50 μg/ml L-ascorbic acid, 10 nM dexamethasone, and 10 mM β-glycerol phosphate) and changed 3 times a week. The experiments were performed for 4 weeks in osteogenic medium. Osteogenic differentiation markers, including alkaline phosphatase (ALP) activity and calcium release were determined by *p*-nitrophenyl phosphate and O-cresolphythalein methods, respectively. The number of cells determined by the fluorometric quantification of cellular DNA was used to normalize the ALP activities and calcium contents.

### Elemental analysis of cell surface cultured scaffolds

Elements, especially calcium (Ca), phosphorous (P), and oxygen (O) on the cell surface after 28-day culture in osteogenic media were analyzed by energy-dispersive X-ray spectroscopy (EDX, Philips Model XP 30 CP, USA). The same cell-seeded constructs used in SEM observations were used for EDX analysis.

### Osteogenic potential of scaffolds in in vivo bioassay

All procedures followed home office guidelines on the scientific use of animals (Scientific Procedures) Act 1986. All animal experiments were conducted in agreement with the Chulalongkorn University Animal Care and Use Committee and with ethical approval from the Institutional Review Board of the Faculty of Medicine, Chulalongkorn University.

Eight-week-old male Wistar rats (275–300 g) were used in this study. The G, GC, and GCD scaffolds (2x11x11 mm^3^) were implanted into the subcutaneous tissue to evaluate the formation of ectopic bone tissue under standard sterile conditions. For each rat, the samples and control were placed into the left and right sides of the back. Gelfoam, a medical device used as hemostatic material for bleeding surfaces, served as a control. The rats were anesthetized by intraperitoneal injection of thiopental sodium (60 mg/kg body weight). The rats were shaved and disinfected with betadine solution and then 70 vol% ethanol. A vertical incision was created down the midline of the back. A 1 cm skin incision was created to form four pockets in the subcutaneous tissue. The scaffolds were inserted subcutaneously away from the incision. The wound was sutured with 4–0 prolene suture and cleaned with betadine solution.

### Histological examination

A total of 48 scaffolds were implanted into 12 rats (4 scaffolds per rat). The rats were divided into 2 groups (4 and 8 weeks). After 4 and 8 weeks of implantation, the rats were sacrificed with an overdose of thiopental sodium. The scaffold implants were histologically analyzed for the formation of ectopic bone tissue by hematoxylin and eosin (H&E) and von Kossa staining. The scaffold samples and surrounding tissues were retrieved, fixed in 10 vol% formalin, and then embedded in a paraffin block. The paraffin-embedded tissues were cut (5 μm thick sections) and followed by staining with H&E to observe collagen formation, cell infiltration, and remaining scaffold. The newly formed collagen and calcium deposition induced by the implant scaffolds was perceived using von Kossa staining. Concisely, the cross-sections of scaffold samples were stained with 5 wt% silver nitrate solution, followed by washing with 5 wt% sodium thiosulphate and double-distilled deionized water. The stained sections were histologically observed and images were taken using an Olympus AX80 Provis microscope (Olympus Ltd., Tokyo, Japan). The experiment was independently duplicated. Each experiment was executed to obtain three samples for each experimental group (*n* = 3).

### Statistical analysis

All statistical analyses were performed using the Statistical Package for Social Sciences (SPSS), version 17.0 (SPSS Inc., Chicago, IL, USA). All the experiments were carried out at least three times with triple replications. Data were expressed as mean ± standard deviation (SD). Differences between the experimental groups were examined using the one-way analysis of variance (ANOVA), with Tukey’s post hoc test if ANOVA showed significance. *P*-values < 0.05 were considered statistically significant.

## Results

### Characteristics of the scaffolds

**Fig. 2:**
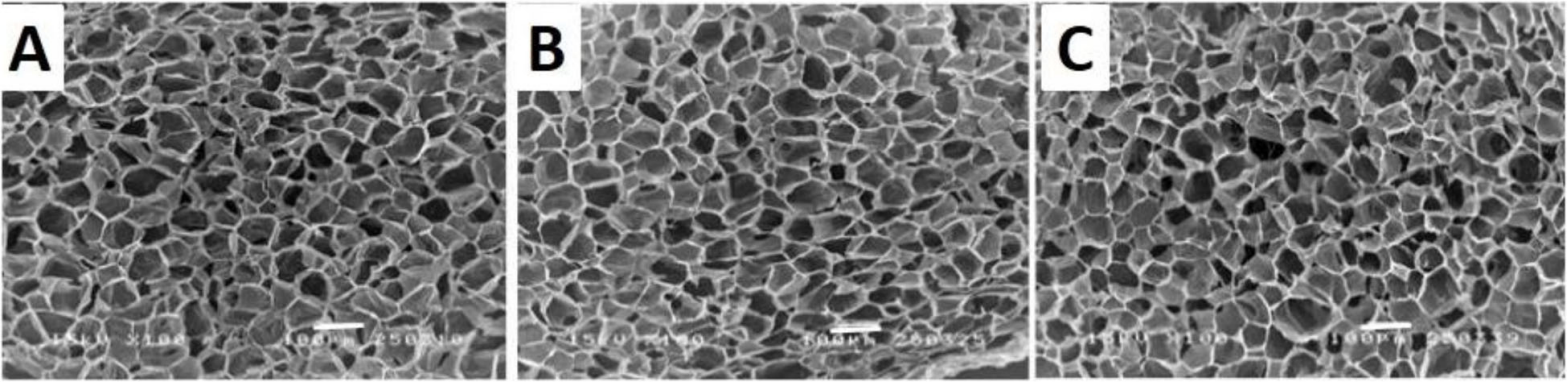
Scanning electron micrographs showing cross-sectional images of the scaffolds. **A** Gelatin (G) scaffolds **B** gelatin/chitooligosaccharide (GC) scaffolds **C** gelatin/chitooligosaccharide/demineralized bone matrix (GCD) scaffolds. Porous, interconnected structure can be evident. (scale bar = 100 μm)

The cross-sectioned microstructures of the G, GC, and GCD scaffolds are displayed in Fig. 2. The physicochemical properties of G, GC, GCD scaffolds are illustrated in Table 1. All scaffolds exhibited a porous interconnected structure. Pore sizes of the scaffolds ranged from 90 to 125 μm with 83–91% porosity. The gelatin scaffolds possessed the greatest pore size (125.3 μm) and the most homogeneous pore distribution. The GC and GCD scaffolds exhibited a homogeneous fibrous structure with smaller pore sizes than the gelatin scaffolds. The addition of COS and DBM on gelatin scaffolds significantly decreased pore size and porosity but enhanced the compressive modulus of the scaffolds. The GCD scaffolds revealed significantly greater compressive modulus than the G and GC scaffolds. The swelling ratio of all scaffolds ranged approximately from 4 to 10 mg water per mg dry scaffold.

**Table 1:** Characteristics of G, GC, and GCD scaffolds

Scaffolds	Pore size (μm)	Porosity (%)	Compressive modulus of wet scaffold (kPa)	Swelling ratio (mg water/mg dry scaffold)
G	125.3 ± 12.5	90.6 ± 2.1	36.0 ± 7.2	10.1 ± 0.4
GC	103.2 ± 11.7*	88.2 ± 1.4	45.0 ± 5.6	6.7 ± 0.7*
GCD	91.7 ± 14.3*	82.9 ± 1.8*	51.0 ± 5.1*	4.0 ± 0.8*

*P < 0.05, significant against the value of gelatin scaffolds

### Attachment and proliferation of MSCs cultured on the scaffolds

**Fig. 3:**
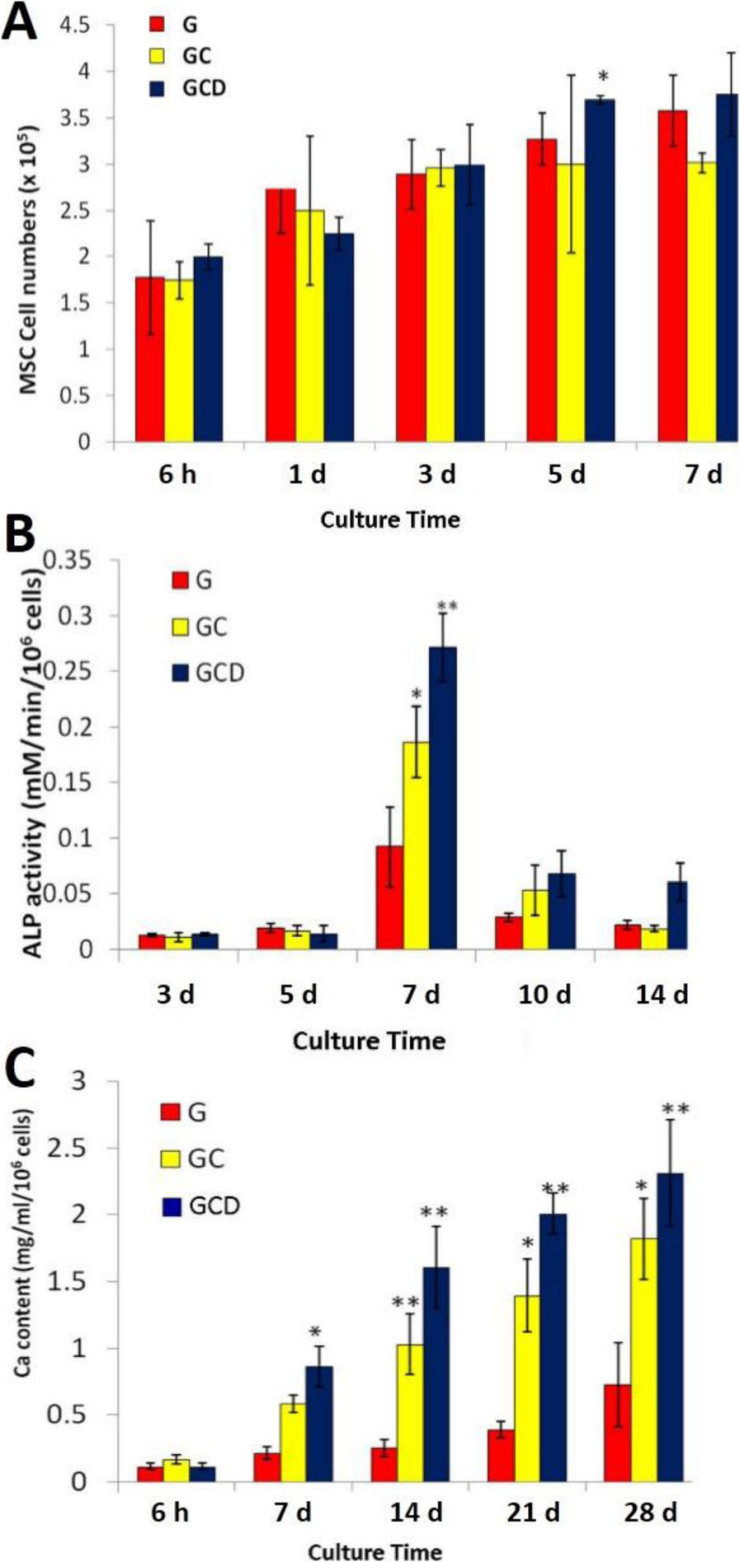
Biological characterization of human MSCs cultured on the G, GC, and GCD scaffolds. **A** Number of MSCs attached and proliferated in G, GC, and GCD scaffolds cultured in α-MEM + 15% FBS for 1, 3, 5, and 7 days **B** ALP activity of MSCs in the scaffolds for 3, 5, 7, 10, and 14 days **C** Calcium content of MSCs in the scaffolds for 7, 14, 21, and 28 days. The results are shown as the mean ± SD (*n* = 5). * and ** represented significant difference relative to G scaffolds within the same culture period at *P* < 0.05 and *P* < 0.001, respectively

The numbers of attached and proliferated MSCs on the G, GC, and GCD scaffolds when cultured in proliferating medium for 6 h, 1, 3, 5, and 7 days are presented in Fig. 3a. It was observed that MSCs could attach and proliferate on GC and GCD scaffolds to a similar extent to those on the pure gelatin scaffold. Furthermore, the number of cells on these scaffolds slightly increased during the period of culture time. The number of MSCs cultured on GCD scaffolds tended to be greater than that of other groups after 5 days of the culture. Therefore, it could be implied that the scaffolds prepared from gelatin, COS, and DBM could support cell activities.

### Osteogenic differentiation of MSCs cultured on the scaffolds

Figure 3b demonstrates ALP activity of MSCs cultured on the scaffolds in osteogenic medium. For any nature of scaffolds, ALP activity of MSCs was greatest after 7 days of the culture and declined thereafter. A high production of ALP activity was evident for MSCs cultured in the GCD scaffolds. The calcium content MSCs cultured on the scaffolds in the osteogenic medium is illustrated in Fig. 3c. Along the culture period, calcium production elevated for all scaffolds. After 7 days of culture, MSCs cultured on the GCD scaffolds revealed greater calcium production than those cultured on G and GC scaffolds. The GCD scaffolds tended to increase calcium content by MSCs better than G and GC scaffolds. The highest calcium production was observed after 28 days of the culture. The GCD scaffolds exhibited the maximum amount of calcium content.

### Calcium phosphate by MSCs cultured on the scaffolds

**Fig. 4:**
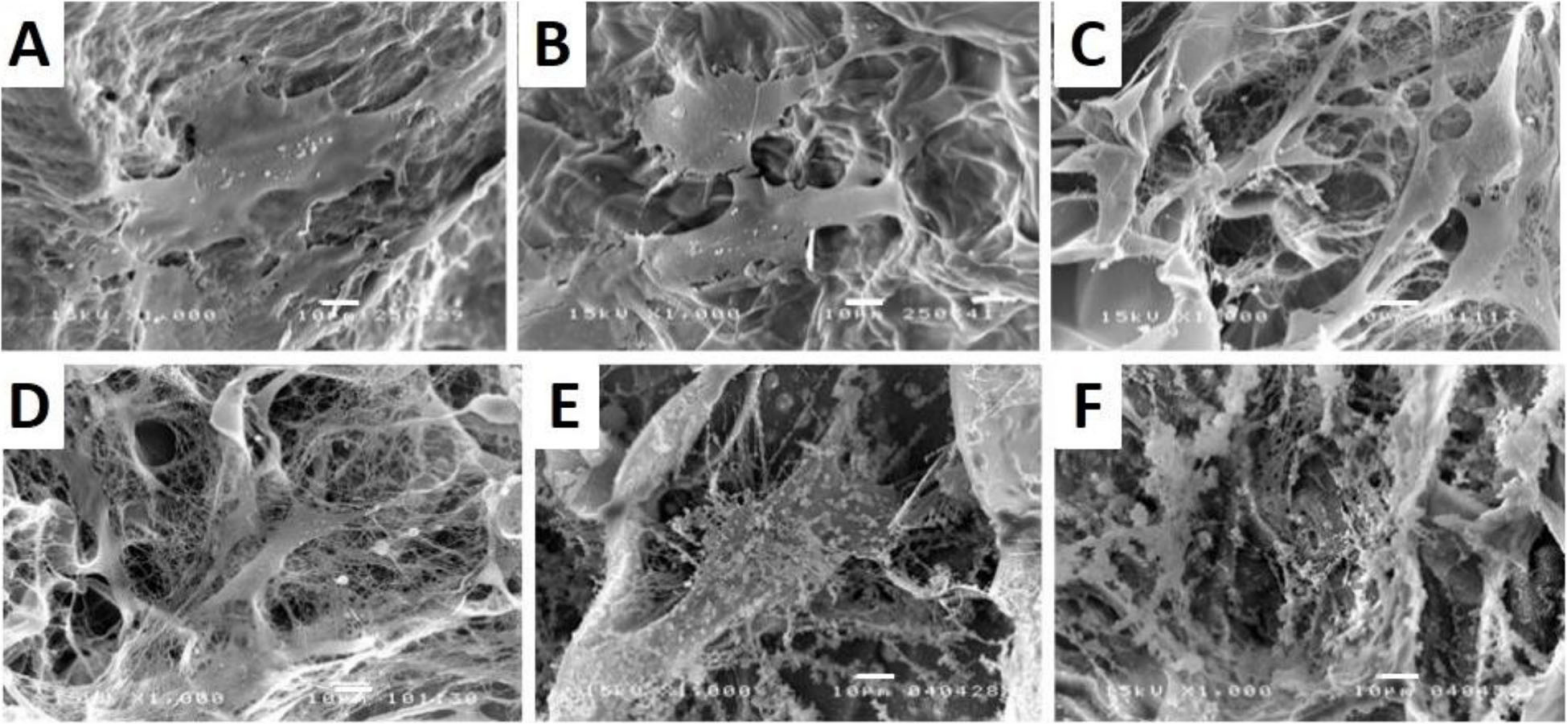
Scanning electron micrographs of cell-seeded scaffolds. MSCs were cultured in proliferating medium for 7 days (**A**-**C**) and 28 days of culturing showing the cellular attachment and spreading (**D**-**F**). **A**, **D** G scaffolds **B**, **E** GC scaffolds **C**, **F** GCD scaffolds. (scale bar = 10 μm)

The morphology of MSCs cultured on the scaffolds in the osteogenic medium for 7 and 28 days was examined using SEM (Fig. 4). Spread MSCs covered the scaffold surface after 7 and 28 days, regardless of scaffold types. Moreover, calcium phosphate deposition was constituted homogenously on the scaffold surface after cultured for 28 days, particularly the GC and GCD scaffolds. It was discerned that MSCs cultured in the GC and GCD scaffolds were more widely spread and produced a greater degree of the fibrous extracellular matrix than those in the G scaffolds. The GCD scaffolds tended to promote a higher density of calcium phosphate nodules than the G scaffolds. Subsequently, EDX analysis of the scaffolds before and after MSCs in osteogenic culture for 28 days was executed. Table 2 displays the quantitative weight percentage of elements including calcium and phosphate on the scaffolds. The findings unveiled that calcium phosphate was not detected in the scaffolds without MSCs culture (Table 2). The GC and GCD scaffolds exhibited significantly greater content of calcium phosphate than the G scaffolds. The greatest percentages of calcium and phosphate were observed in the GCD scaffolds. The highest values of calcium:phosphate ratio (1.7–1.8) were identified for the GCD scaffolds. The order of calcium phosphate percentage in the scaffolds was GCD > GC > G scaffolds. This order corresponded to that of the calcium content of MSCs cultured on the scaffolds (Fig. 3c).

**Table 2:** EDX analyses of G, GC, and GCD scaffolds before and after culture with MSCs in osteogenic medium for 28 days

Type of scaffolds	Wt% (without MSCs)						Wt% (with MSCs)					
	O	Na	Mg	Ca	P	Ca: P	O	Na	Mg	Ca	P	Ca: P
G	81–85	9–15	5–18	0	2–4	0	56–61	7–9	1–2	10–13	20–24	0.5–0.6
GC	77–88	4–8	3–8	0–1	3–5	0–0.2	27–41	5–7	4–7	19–34	16–23	1.2–1.5
GCD	68–83	5–13	7–18	1–2	3–5	0.3–0.4	60–75	5–10	3–5	20–44	12–25	1.7–1.8

### In vivo bioassay of ectopic bone formation induced by each scaffold

**Fig. 5:**
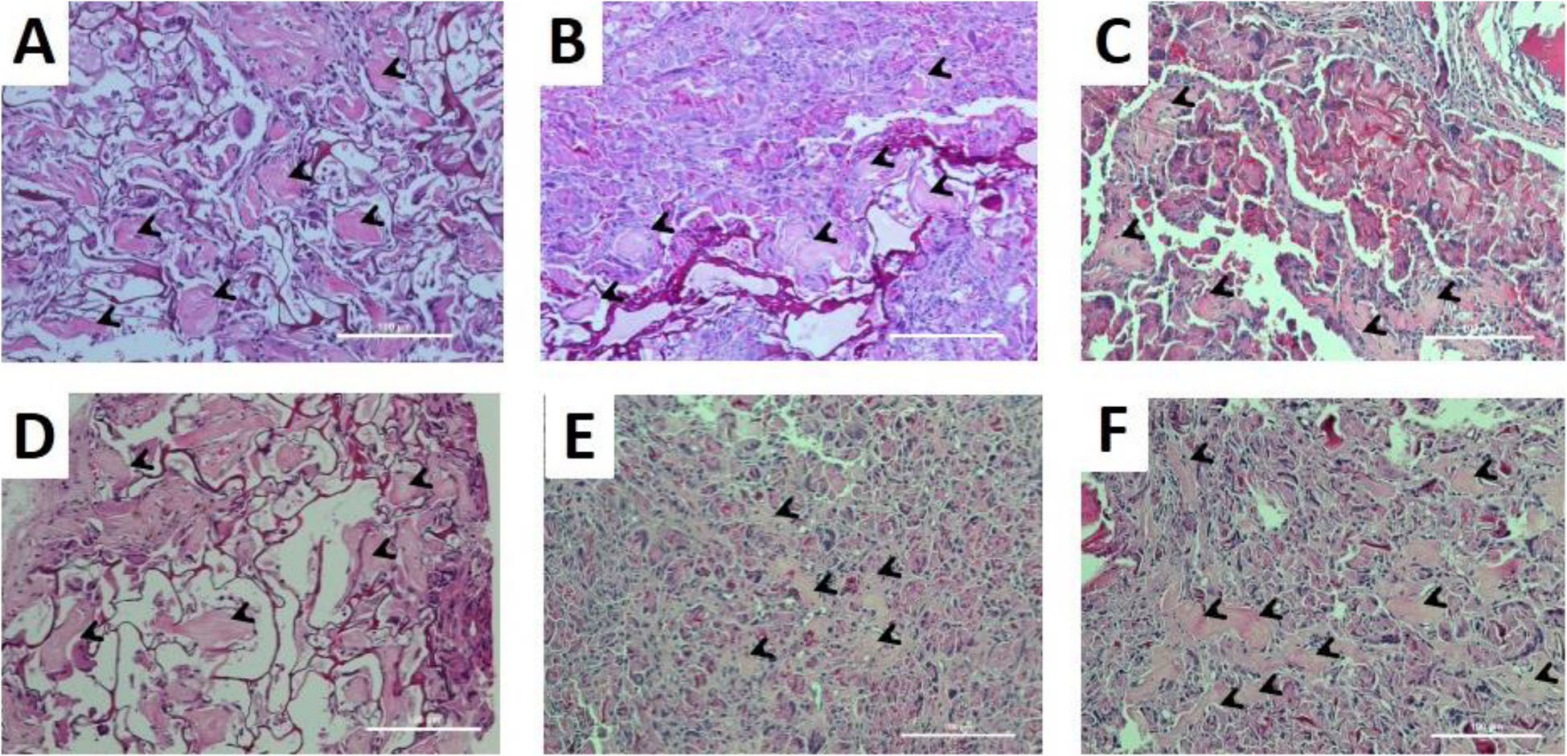
Histological evaluation of constructs subcutaneous implantation. Representative H&E stained histological sections of scaffolds after 4 weeks implantation (**A**-**C**) and 8 weeks implantation (**D**-**F**) of G, GC, and GCD scaffolds. **A**, **D** G scaffolds **B**, **E** GC scaffolds **C**, **F** GCD scaffolds. Collagen matrix and high cell infiltration were observed, particularly more intense in the GC and GCD scaffolds. Arrows indicate osteoid formation. (scale bar = 100 μm)

**Fig. 6:**
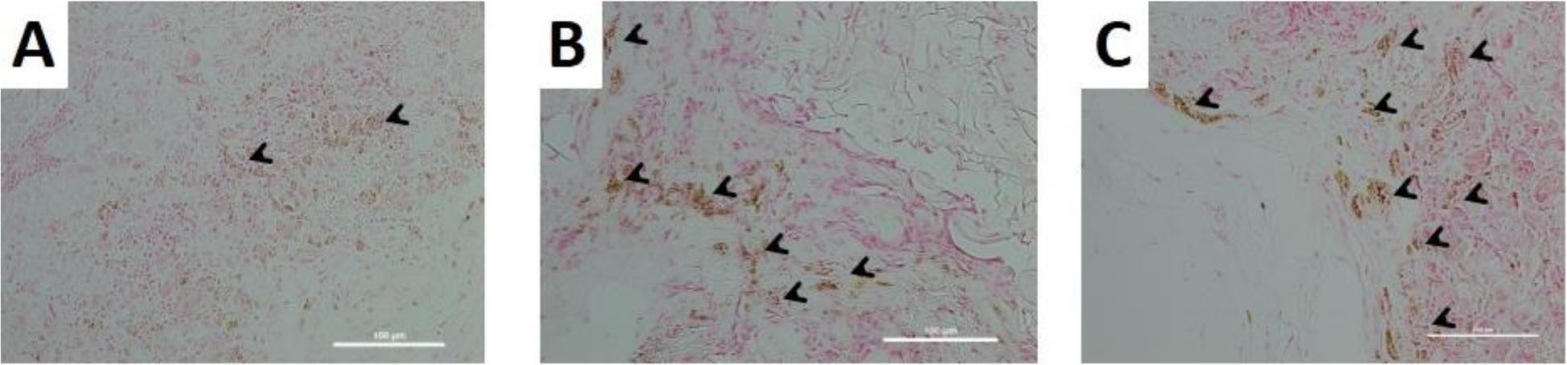
Representative von Kossa histological sections of subcutaneously implanted G, GC, and GCD scaffolds in Wistar rat at 8 weeks post implantation. **A** G scaffolds **B** GC scaffolds **C** GCD scaffolds. Arrows indicate positive calcium deposition and the synthesized mineralized matrix (dark brown staining). (scale bar = 100 μm)

With regard to the remarkable capability to stimulate in vitro osteogenic differentiation of MSCs, the G, GC, and GCD scaffolds were chosen for subsequent in vivo Wistar Rat study. After 4 and 8 weeks of implantation, the ectopic bone formation of the GC and GCD scaffolds were compared to that of the G scaffolds. Figures 5 and 6 depict histological evaluation regarding the in vivo ectopic bone formation of each scaffold implanted. Four weeks after implantation, histological sections illustrated new collagen formation with high cell infiltration inside the GC and GCD scaffolds. The G scaffolds had a low amount of extracellular matrix surrounding very few cells, whereas a greater number of infiltrated cells were evident in the GC and GCD scaffolds. At 8 weeks following implantation, the GC and GCD scaffolds had greater cell density and cells surrounded by scaffolds and extracellular matrix. Moreover, cells were homogeneously distributed, and *osteoid*-*mimicking* dense collagen was more pronounced, especially in the GCD scaffolds. As mineralization is also a valuable marker of bone tissue formation, von Kossa staining of the implanted scaffolds was performed to observe the presence of tissue mineralization (Fig. 6). This stain resulted in brown, opaque spots when calcium deposits were encountered. It is discernable in von Kossa staining that calcium was accumulated around the edge of GC and GCD scaffolds after 8-week implantation. Calcium was homogeneously assembled, principally on the surface of the GCD scaffolds. On the other hand, no calcium accumulation was observed in the G scaffolds.

## Discussion

Even though several approaches have been introduced to accelerate new bone formation, suitable therapeutic strategies to regenerate a new bone with optimal morphology and mechanical features have not yet been instituted. Tissue engineering has been used to reconstruct bone substitutes for the healing of extensive bone defects. Cells, composite scaffolds, and signaling molecules are the three essential components for the construction of substituted tissues that repair, replace, or regenerate diseased and damaged tissues or organs [1]. Natural polymers, such as gelatin and COS, have been utilized in fabricating scaffolds in bone tissue engineering [2]. COS is a low molecular weight water-soluble chitosan developed by enzymatic or chemical hydrolysis of chitosan. Furthermore, COS is noncytotoxic, biodegradable, and shows superior biological properties to the high molecular weight chitosan [10]. Gelatin/COS scaffolds demonstrate a seemly microenvironment for cell attachment and proliferation [10]. The addition of growth factors within scaffolds can enhance the osteoconductive and osteoinductive characteristics and, therefore, improve new bone regeneration. DBM serves as an attractive scaffold applicable for osteoinduction in bone tissue engineering and osteoconductive network of collagen supports attachment and migration of cells. The present research highlights developing a novel tissue engineering scaffold comprising of gelatin, COS, and DBM and exploring whether it could be a candidate for application in bone tissue engineering.

In the current study, we fabricated G, GC, and GCD scaffolds. The morphology and internal structure of the three scaffolds were comparable. All scaffolds exhibited proper pore size (> 90 μm) with high porosity (> 80%), which could be advantageous for the transport of nutrients and oxygen as well as promote the adhesion and proliferation of cells [16]. A previous study disclosed that the appropriate pore size for bone growth was approximately 80–100 μm [17]. The porosity and interior open-pore framework are beneficial features for biomaterials aimed for use in bone tissue engineering since they allow cell ingrowth as well as nutrient diffusion and act as an essential part in cell migration and proliferation [18]. The GCD scaffolds present a suitable pore size supporting a decent microenvironment for bone regeneration and repair.

According to the mechanical features, the GCD combination improved the stiffness of the scaffolds, potentially attributing to their condensed pore thickness and the enlarged scaffolding density. In fact, scaffolds synthesized from inorganic biomaterials are mechanically better than organic scaffolds without the addition of inorganic substances. The DBM used in the GCD scaffolds contains a minimal amount of calcium (as evident in EDX analysis), leading to the increased compressive modulus of the GCD scaffolds.

All three scaffolds provided suitable cell attachment because they possessed high porosity and supported cells to contact the pore walls. When cultured for 1, 3, 5, and 7 days, the cells in the GCD scaffolds were more active than those in the G and GC scaffolds. The findings of cell proliferation uncovered DBM as a suitable biomaterial for MSCs. DBM mainly comprises collagen type I and growth factors, including BMPs, which impart osteoconductivity and osteoinductivity to the scaffolds. In the study, the cell viability in the scaffolds was appraised by DNA quantification assay. The DNA amount in cell/scaffolds was fluorometrically quantified by using Hoechst 33258 dye. Several metabolic activity assays, including MTT, MTS, and CCK-8 may not accurately reflect cell proliferation in scaffold applications or at high cell densities in three-dimensional cultures. It is prudent that metabolic assays varied greatly with cell density and do not necessarily correlate linearly with increasing cell number and activity over time in culture [19]. In contrast, DNA quantification assay using the fluorescent dye bisBenzimide (Hoechst *33258*) is an ultrasensitive DNA in vitro assay that exhibits maximum fluorescence enhancement, low inter- and intra-assay variability, and no interference with extracellular matrix [20]. Therefore, DNA content of cells was evaluated to investigate cell attachment and proliferation of MSCs in the scaffolds.

Alkaline phosphatase has been involved in various fundamental roles in bone mineralization in vivo [21]. Increments of alkaline phosphatase activity correlates with the development of osteoblastic lineage. ALP activity has been known to be an early transient biomarker of osteoblast differentiation in MSCs and ordinarily serves as a great parameter of differentiation [22]. Therefore, ALP activity was assessed to examine osteogenic differentiation of MSCs in vitro onto the scaffolds. The three-dimensional cultures showed a peak of ALP production at 7 days of culture, supporting that the GCD scaffolds provide a favorable three-dimensional microenvironment for cell differentiation. The subsequent decline in ALP activity after 7 days corresponds to the process of the mineralization and EDX analysis supports this result. Besides, calcium deposition on the scaffolds is an indicator of the differentiation of MSCs into osteoblasts, which begins early synthesis and mineralization activity [23].

In this study, the highest ALP activity was observed after culturing 7 days in all scaffold samples. The reason for this finding should be that ALP activity is a transient early biomarker of osteoblast differentiation in MSCs, being upregulated initially and downregulated as differentiation progresses. It was demonstrated that ALP was related with calcification and an increased expression of this enzyme was distinctly needed just prior to the onset of matrix mineralization, providing localized enrichment of inorganic phosphate, one of the components of apatite, the mineral phase of bone [24]. Therefore, an initial rise in ALP activity was expected, followed by a decrease allied with further differentiation of the cells, when ALP production slowed. The reduced ALP activity could be attributable to the fact that more cells cultured on the scaffolds stepped into the next differentiation stage. This was along with the intracellular calcium increase which could determine an inhibitory effect. ALP decrease could represent a return to osteoprogenitor cells or maturation to osteocytes, which basically express small quantities of this enzyme. There was a major decline in ALP activity during matrix vesicle mineralization. The decline in ALP activity is very closely concurrent with the rapid accumulation of calcium by matrix vesicles.

The result concerning calcium deposition in culture of MSCs is a biomarker of full maturation. There is a close relationship between the calcium deposition accumulation cultures and ALP activity. This study evinced that a remarkable decline of ALP activity did occur during the matrix vesicle mineralization. The time of onset and the extent of decrease in ALP activity were observed to mirror quite closely the time of onset and the extent of calcium accumulation by the matrix vesicles of all the scaffolds.

Although gelatin/chitosan mixture scaffolds have been widely investigated, information of gelatin/COS/DBM blends is still limited. The capability of these scaffolds to induce new bone formation in vivo was assessed using rat ectopic pouch model. The results revealed that the GC and GCD scaffolds exhibited higher cell density and cells were surrounded by scaffolds and extracellular matrix. Furthermore, cells were extensively distributed throughout the scaffolds and the *osteoid*-*like* matrix was more distinct, particularly in the GCD scaffolds. These findings were supported by von Kossa staining showing calcium accumulation was present around the edge of GC and GCD scaffolds following the 8-week implantation.

Previously, Rattanavaraporn and colleagues have conducted the implantation of G/COS scaffolds into nude mice and demonstrated that it enhanced osteogenic differentiation and calcium deposition [11]. Moreover, collagen scaffolds with incorporated DBM could enhance cell attachment, stimulate cell proliferation, and promote osteogenic differentiation [12]. Additionally, DBM combined with small intestinal submucosa composite exhibited superior osteoinductivity, provided a suitable environment for osteoblast differentiation, and ultimately enhanced new bone formation [25]. Our findings from this study are in accordance with reports from recent studies, although different scaffolds were used. The in vivo rat ectopic pouch osteoinduction model supports that the GCD scaffolds generate cell infiltration, collagen formation, osteoid-like matrix, and calcium mineralization.

It should be denoted that DBM exhibits osteoinductive features, which is why implant groups comprising DBM result in substantiated ectopic bone formation. In ectopic implant aspects, bone regeneration necessitates the active recruitment and initiation of MSCs. The present in vivo investigation advocated an association between the presence of COS, DBM, and new bone regeneration in an ectopic rat model.

However, this study acknowledged some limitations that should be mentioned. First, we could not display the surface SEM images of the scaffolds due to a limited number of scaffold biomaterials. Second, the combination of gelatin and DBM scaffolds were not included in the present study. Future *research* is warranted to *compare* either alone or among various combinations of G, COS, and DBM scaffolds. Another *caveat* from the present study is the lack of immunostaining for osteogenic markers in the in vivo samples. Further immunohistochemical staining of osteogenic markers including osteocalcin, osteopontin, and/or bone sialoprotein could verify the findings and yield more valuable information. Lastly, Masson’s trichrome staining for cross-linked collagen and/or Alizarin red staining for calcium will be needed to confirm the ectopic bone regeneration of the scaffolds following the implantation.

## Conclusion

The combination scaffolds of G, COS, and DBM promoted cell attachment, cell proliferation, and osteogenic differentiation in vitro. The MSCs cultured in the GCD scaffolds demonstrated greater osteogenic differentiation than those cultured in the G and GC scaffolds. Furthermore, the G, GC, and GCD biomaterial scaffolds could support in vivo ectopic new bone formation in in vivo rat model. The GCD scaffolds exhibited maximum osteoinductive capability compared with others and are a promising candidate for bone tissue engineering application. Altogether, the current study has uncovered that the GCD scaffolds present a potential bone graft substitute with osteoconductive and osteoinductive features that would affirm augmented bone formation.

## Data Availability

The datasets during and/or analyzed during the current study available from the corresponding author or reasonable request.

## Abbreviations

ALP: Alkaline phosphatase
α-MEM: Alpha-minimum essential medium
ANOVA: Analysis of variance
bFGF: Basic fibroblast growth factor
BMPs: Bone morphogenetic proteins
Ca: Calcium
COS: Chitooligosaccharide
DBM: Demineralized bone matrix
EDX: Energy-dispersive X-ray spectroscopy
FBS: Fetal bovine serum
G: Gelatin
GAG: Glycosaminoglycan
GC: Gelatin/chitooligosaccharide
GCD: Gelatin/chitooligosaccharide/demineralized bone matrix
H&E: Hematoxylin and eosin
MSCs: Mesenchymal stem cells
O: Oxygen
P: Phosphorous
RGD: Arginine-glycine-aspartic acid
SEM: Scanning electron microscopy
SSC: Sodium citrate-buffered saline solution
SPSS: Statistical Package for Social Sciences
TGF-β: Transforming growth factor-β
